# Expression Sites of Colligin 2 in Glioma Blood Vessels

**DOI:** 10.1111/j.1750-3639.2008.00248.x

**Published:** 2010-01

**Authors:** Dana Mustafa, Marcel van der Weiden, PingPin Zheng, Alex Nigg, Theo M Luider, Johan M Kros

**Affiliations:** 1 Departments of Pathology, Laboratory of Neuro-oncology and Clinical Proteomics, Erasmus Medical CenterDr. Molewaterplein 50, 3015 GD Rotterdam, The Netherlands; 2 Departments of Neurology, Laboratory of Neuro-oncology and Clinical Proteomics, Erasmus Medical CenterDr. Molewaterplein 50, 3015 GD Rotterdam, The Netherlands

**Keywords:** αSMA, collagen I, collagen IV, colligin 2, endosialin, glioma, HSP47, neoangiogenesis, NG2

## Abstract

In a previous study using state-of-the-art proteomic techniques, we identified colligin 2 (HSP47) as a glioma blood vessel-specific protein. In the present study we precisely localized the expression of colligin 2 in the blood vessels of diffusely infiltrating gliomas and relate the expression to the distinct cellular components of the vessels by using multiple immunolabeling and confocal microscopy. We grouped the glioma blood vessels into morphological categories ranging from normal looking capillaries to vessels with hypertrophic and sclerotic changes. The expression patterns of various markers of endothelial and pericytic differentiation were correlated with the position of the cells in the vessels and the expression of colligin 2. We found that colligin 2 is expressed in all categories of glioma blood vessels in cells with endothelial and pericytic lineage. Expression of colligin 2 was also found in cells scattered around blood vessels and in few glial fibrillary acidic protein-positive cells within the blood vessels. There is overlap in the expression of colligin 2 and the collagens type I and IV for which colligin 2 is a chaperon. We conclude that colligin 2 is expressed in all cellular components of glioma blood vessels and may serve as a general marker for active angiogenesis.

## INTRODUCTION

Gliomas are among the neoplasms with the highest degree of vascularization (19). These neoplasms contain increased numbers of blood vessels relative to normal brain tissue, and the vessel walls are variably thickened because of proliferation of their cellular constituents (5). Along with glial tumor progression, normal-looking blood vessels gradually hypertrophy into glomeruloid structures with multiple lumina ultimately degenerating into end-stage vessels with sclerotic walls and obliterated lumina (12). Many vessels become prone to thrombosis and recanalization of organized thrombi is a frequently observed phenomenon. The newly formed blood vessels are leaky because of the defective and aberrant basal membrane formation (24). It has long been appreciated that tumor growth and progression are dependent on angiogenesis, but the elucidation of the molecular mechanisms that trigger the formation of new blood vessels is still in its early stage (3). Although many aspects of the angiogenic switch, that is, the transition of dormant pre-existing blood vessels into an actively sprouting vasculature are not unravelled yet, a variety of angiogenic regulators have been detected (37) and some have already been tested for the development of anti-angiogenic therapies (25). Besides the therapeutic approach aiming at destroying glioma vasculature, attempts to normalize the structure and function of the newly formed and dysfunctional blood vessels are also undertaken for reaching better penetration of chemotherapeutics and optimize conditions for effective radiation therapy (8, 15). In order to manipulate the cerebral microcirculation, knowledge of the interplay of the cells involved and of the underlying molecular mechanisms is required (31).

The cells in blood vessel walls are characterized either by their position relative to the vascular lumina and/or by their immunohistochemical profiles. In normal blood vessels, endothelial cells are considered to line the lumina and express CD31 (4), CD34 (27), Von Willebrand factor (36) and more. Pericytes (also indicated as smooth muscle cells or mural cells) form an incomplete layer around the endothelial cells (32). CD105 is a marker for activated endothelial cells taking part in neoangiogenesis not only in gliomas but also in other tumors (13, 20, 23). Most available data on the cells involved in neoangiogenesis concern endothelial cells and factors regulating their proliferation (11, 16). In recent studies the importance of pericytes and their interaction with endothelial cells for blood vessel formation, stabilization and function was highlighted (28, 33). There are indications of the existence of various subtypes of pericytes in various organs with different functions and locations in the vessels (2, 34). Immunohistochemical marker profiles for pericytes are diverse and vary between organs and developmental stages (2). Because of this diversity, no general pan-pericytic marker is known (3). A well-known marker for pericytes in cerebral vasculature is α-smooth muscle actin (αSMA) (32). The marker NG2 is also used for staining of brain pericytes (7) and has been instrumental in proving that pericytic precursor cells are recruited to sites where vessel growth and repair are occurring (6). Recently, endosialin was found to be strongly upregulated in pericytic cells in the developing human brain (33) and glioma (28). Its expression is closely associated with other perivascular cells (28). Other markers used to identify pericytes include platelet-derived growth factor receptor beta (PDGFR-β) (33), CD13 and desmin (35), but none is specific for these cells.

In a previous study using state-of-the-art proteomics techniques, colligin 2 was identified as a protein that is expressed in glioma neovasculature but not in normal brain vasculature (21). In addition, we found that expression of colligin 2 is not limited to glioma vasculature, but is also seen in vasculature of non-glial tumors. Further, its expression is found in non-neoplastic tissues, but only under circumstances of active angiogenesis like wound healing and recanalization of thrombi. Colligin 2 protein (also known as collagen binding stress protein; HSP 47) is localized in the endoplasmic reticulum and specifically binds to collagen type I, collagen type IV and gelatin (14). It assists in the formation of the rigid triple-helical structures of collagen type I (22) and contributes to the maturation of collagen type IV (18). Collagen is the major component of the basement membrane and a crucial element of the blood–brain barrier (BBB) (1, 9, 30). The aim of the present study was to precisely localize the expression of colligin 2 in the blood vessel walls of gliomas and associate the expression to the specific cellular components of the vessel walls in order to obtain indications as to the role of this protein in glioma angiogenesis. To this end, in addition to conventional microscopy of adjacent slides, confocal microscopy was used because of the superior levels of resolution and creating three-dimensional representations.

## MATERIALS AND METHODS

### Patients and tumor samples

Twenty glioblastoma (GBM) samples were taken from the files of the Department of Pathology, Erasmus MC, Rotterdam. In addition, seven samples of autopsy brains of patients without brain tumors were used as controls. Post-mortem times of the control cases were 8 h or less. For immunohistochemical staining, paraffin embedded and fresh-frozen samples were used depending on the specifications of the antibodies and type of staining. For application of confocal microscopy, fresh-frozen samples were used. The number of samples, clinical data and tumor types are summarized in Table 1.

**Table 1:** Clinical data for the patient that had been used in this study. Abbreviations: ARDS = adult respiratory distress syndrome; AVM = arteriovenous malformation; F = frontal; GBM = glioblastoma; LE = left; P = parietal; RI = right; SAB = subarachnoidal hemorrhage; T = temporal.

High-grade diffusely infiltrating glioma				
Glioma samples	Sex	Age (years)	Tumor site	Diagnosis
T1	Female	30	Ri F	GBM
T2	Female	30	Ri F	GBM
T3	Female	54	Le F-P	GBM
T4	Female	58	Le F-P	GBM
T5	Female	63	Le F	GBM
T6	Male	36	Le F	GBM
T7	Male	44	Multifocal	GBM
T8	Male	46	Le T	GBM
T9	Male	47	Le T	GBM
T10	Male	47	Ri T-P	GBM
T11	Male	48	Le F	GBM
T12	Male	51	Ri F	GBM
T13	Male	55	Ri F	GBM
T14	Male	56	Le T-P	GBM
T15	Male	57	Le T	GBM
T16	Male	57	Ri F	GBM
T17	Male	57	Le T	GBM
T18	Male	62	Le T	GBM
T19	Male	62	Le T	GBM
T20	Male	68	Le F	GBM

Controls				

Normal samples	Sex	Age (years)	Location	Diagnosis

S1	Female	39	Ri F	SAB
S2	Female	48	Ri F	SAB
S3	Female	60	Ri F	Pneumonia
S4	Female	76	Ri F	Pneumonia
S5	Male	31	Ri F	AVM
S6	Male	34	Ri F	Hemorrhage brain stem
S7	Male	70	Ri F	ARDS

### Immunohistochemistry

#### Single-staining procedures

Twelve biopsy samples of GBMs and five autopsy control samples were used. The samples were collected in a consecutive way. From each paraffin embedded sample, adjacent slides of 5 µm sections were stained with various antibodies. Because NG2 and CD105 antibodies do not work on paraffin embedded tissues, these and the other markers were all used on adjacent frozen sections. Immunohistochemical staining was performed following the manufacturer's instructions (alkaline phosphatase technique). The antibodies and their specifications are summarized in Table 2. Briefly, the paraffin sections were mounted onto poly-L-lysin-coated slides, deparaffinized in xylene for 15 minutes and rehydrated through graded alcohol and washed with water. Frozen sections were fixed in acetone for 15 minutes and then air-dried. The sections were washed with phosphate-buffer saline (PBS) and incubated with the antibody for 30 minutes. After washing the sections with PBS, the corresponding antigen was added and incubated 30 minutes at room temperature. New Fuchsin alkaline phosphatase substrate solution was freshly prepared and the sections were incubated for about 30 minutes. Afterwards, the sections were washed with tap water, counterstained and coverslipped with permanent mounting medium.

**Table 2:** Antibodies used in this study.

Name	Dilution	Commercial source
Monoclonal colligin 2	1:500	Stressgen, Michigan, USA
Polyclonal colligin 2	1:100	MBL international, Woburn, Canada
CD31	1:40	Dako, Glostrup, Denmark
CD34	1:30	Dako, Glostrup, Denmark
CD105	1:2000	Dako, Glostrup, Denmark
NG2	1:100	ZYMED laboratories, California, USA
Endosialin	1:500	Prof. Isacke, Institute of Cancer Research, London, UK
αSMA	1:40	Biogenex, California, USA
Collagen I	1:100	Abcam, Cambridge, UK
Collagen IV	1:25	Dako, Glostrup, Denmark
Mib-1 (Ki-67)	1:10	Dako, Glostrup, Denmark
GFAP	1:100	Dako, Glostrup, Denmark
Goat-anti-mouse-CY3	1:100	BioLegend, California, USA
Biotin-horse-anti-mouse	1:200	Vector, Peterborough, UK
Avidin-FITC	1:50	Jackson Immunoresearch, Pennsylvania, USA

#### Double staining procedures

Double immunolabelings were performed combining colligin 2 antibody with the various markers for endothelial cells and pericytes. Ten frozen biopsy samples of GBMs were used for confocal laser microscopy. Adjacent slides of 5-µm sections were mounted onto non-coated microscope slides, fixed in acetone for 15 minutes and then air-dried. Sections were incubated with colligin 2 polyclonal antibody for 30 minutes, washed and incubated again with Cy3-conjugated goat anti-rabbit for 30 minutes. After washing, sections were incubated with the second monoclonal antibody for 30 minutes followed by 30 minutes of labeling with biotin–horse–anti-mouse antibody. Detection was performed by fluorescein isothiocyanate (FITC)-conjugated avidin antibody. Nuclei were counterstained with 4′,6-diamidino-2-phenylindole (DAPI) in a vector sheet (1:1000) and slides were covered and examined with the confocal laser microscope. For all antibodies the staining was always performed single on each sample also, to control for the accuracy of the staining and specificity of the antibodies used. For each antibody, negative controls including the secondary antibodies only were obtained for both single- and double-stained slides. The specifications of the antibodies are summarized in Table 2.

#### Confocal laser-scanning microscopy

Using the confocal microscope allowed the staining and samples with two different antibodies. In addition, the resolution of the confocal microscope and its ability to produce three-dimensional optical images enabled specific targeting the cellular components of glioma blood vessels in a more accurate and reliable way. Confocal images were obtained using a confocal laser-scanning microscope (LSM510; Carl Zeiss Microimaging Inc., Jena, Germany) equipped with a Plan-Neofluar 40×/1.3 NA oil objective (Carl Zeiss Microimaging Inc.). A diode laser was used for excitation of DAPI at 405 nm, an argon laser for FITC at 488 nm and a HeNe-laser for Cy3 at 543 nm. For DAPI an emission bandpassfilter of 420–480 nm was used, for FITC a bandpassfilter of 500–530 nm and for CY3 a longpassfilter of 560 nm. The signals were recorded sequentially (multitrack option) to avoid interference and stored in separate channels.

## RESULTS

### Immunohistochemistry

#### Normal brain

**Figure 1:**
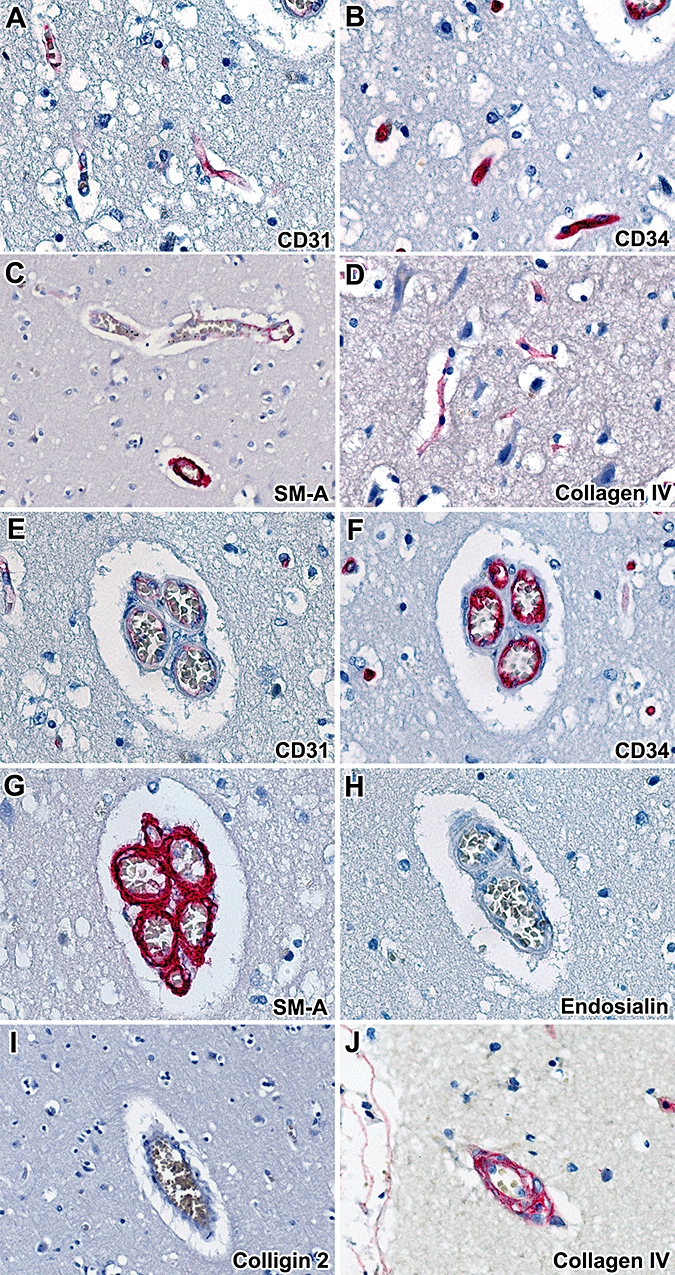
*Expression of the various markers in normal brain*. **A–D.** CD31, CD34 and collagen IV are all expressed in capillaries. αSMA is variably expressed (panels **A, B,D**: ×40; panel **C**: ×20). **E–J.** CD31, CD34, αSMA, collagen IV are expressed in small vessels in normal brain; endosialin and colligin 2 are not expressed (**E,F–H,J**: ×40; **I**: ×20).

The endothelial cells of small and medium-sized vessels in the samples of a normal brain showed immunopositivity for CD31 and CD34 but not for αSMA (Table 3). The mural cells were variably positive for αSMA. None of the cells of the blood vessels in normal brain were positive for CD105, NG2, endosialin or colligin 2 (Figure 1 and Table 3).

**Table 3:** Immunostaining of blood vessels in a normal brain.

Layers in normal cerebral blood vessels	CD31	CD34	CD105	NG2	Endosialin	αSMA	Colligin 2
Endothelial cells (lumina-lining)	+	+	−	−	−	−	−
Pericytes (abluminal lining)	−	−	−	−	−	+/−	−

#### Glioma

**Figure 2:**
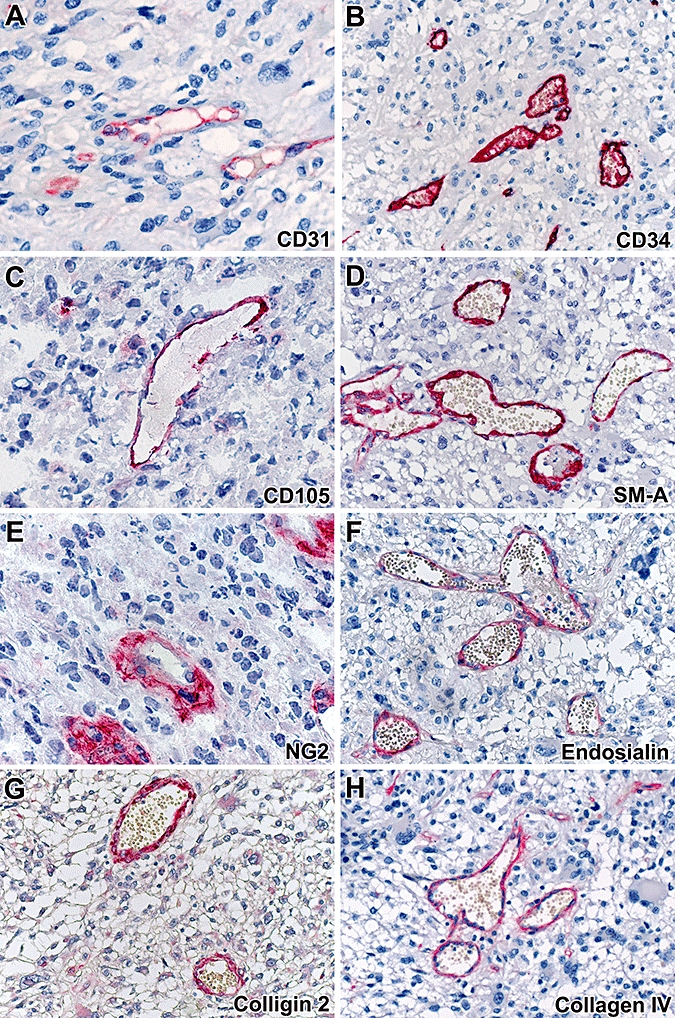
*Expression of the various markers in capillaries and small vessels in glioma*. All capillaries and small vessels are invariably positive for the markers indicated (panels **A,C,E**: ×40; **B,D,F–H**: ×20).

**Figure 3:**
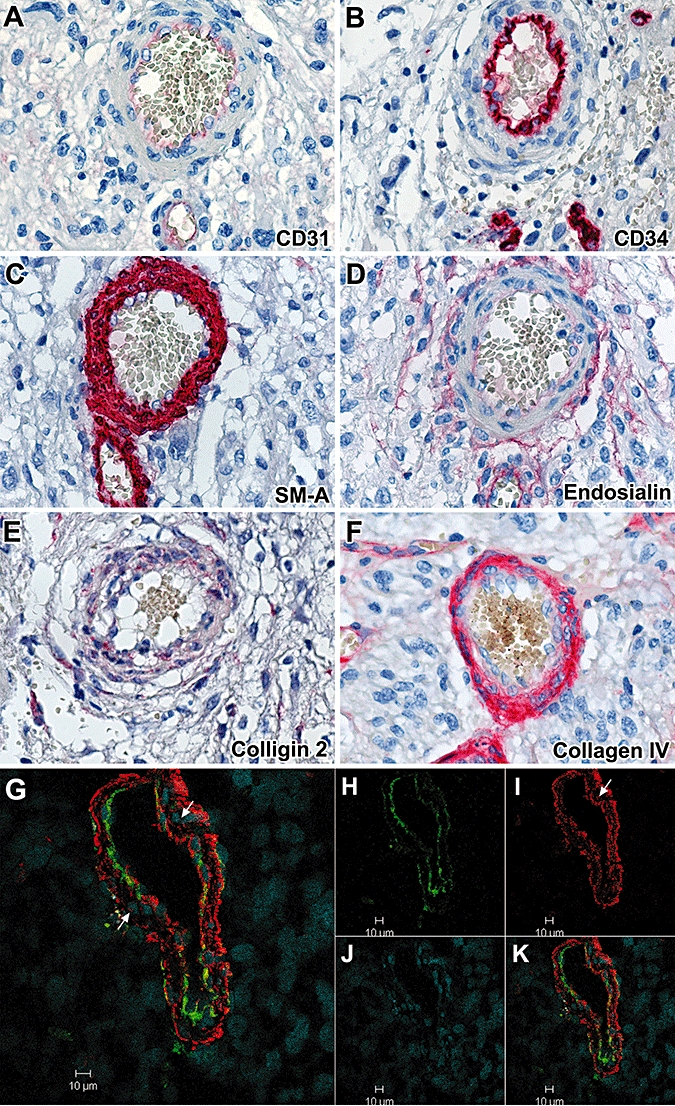
*Expression of the various markers in layered hypertrophic blood vessels in glioma. CD31 and CD34 are expressed in the endothelial layer*. CD105 and endosialin are expressed in the endothelium and the external layer. αSMA, NG2 and collagen IV are expressed in the intermediate and external layers, not in the endothelium. Colligin 2 is expressed in all layers of the vessels (panels **A–F**: ×40). **G–K.** Confocal images of layered hypertrophic blood vessels in glioma for the expression of CD105. Double immunolabeling for CD105/colligin 2. The endothelial cells and the external cells express both CD105 and colligin 2, while the intermediate cells express colligin 2 only (arrows) (panel **H**: green = CD105; panel **I**: red = colligin 2; panel **J**: blue = DAPI; panel **K**: merged picture).

**Figure 4:**
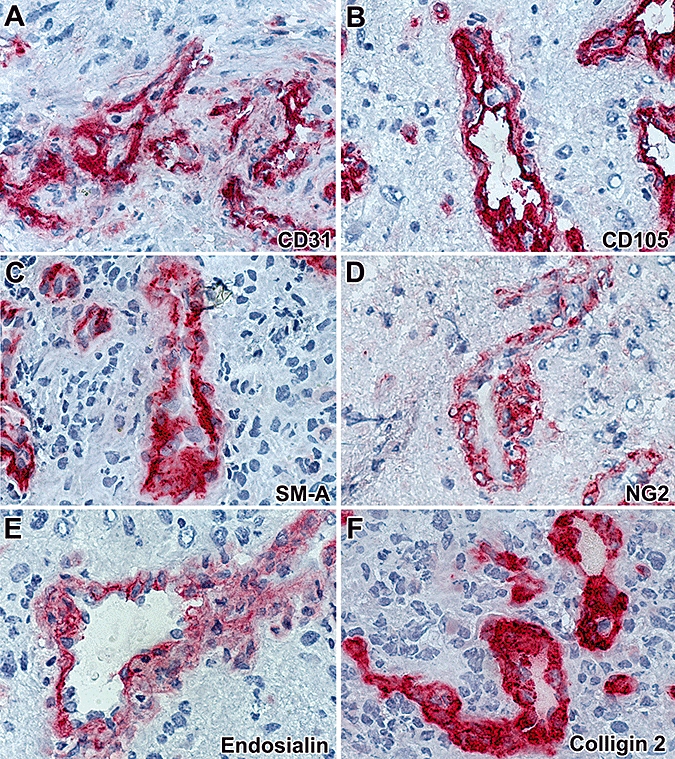
Expression of the various markers in hypertrophic vessels without layered structure. CD31 is exclusively expressed in the endothelial layer; CD105 and colligin 2 are expressed in all layers. SM-A, NG2 and endosialin are present in all layers except the endothelial layer (all panels ×40).

**Figure 5:**
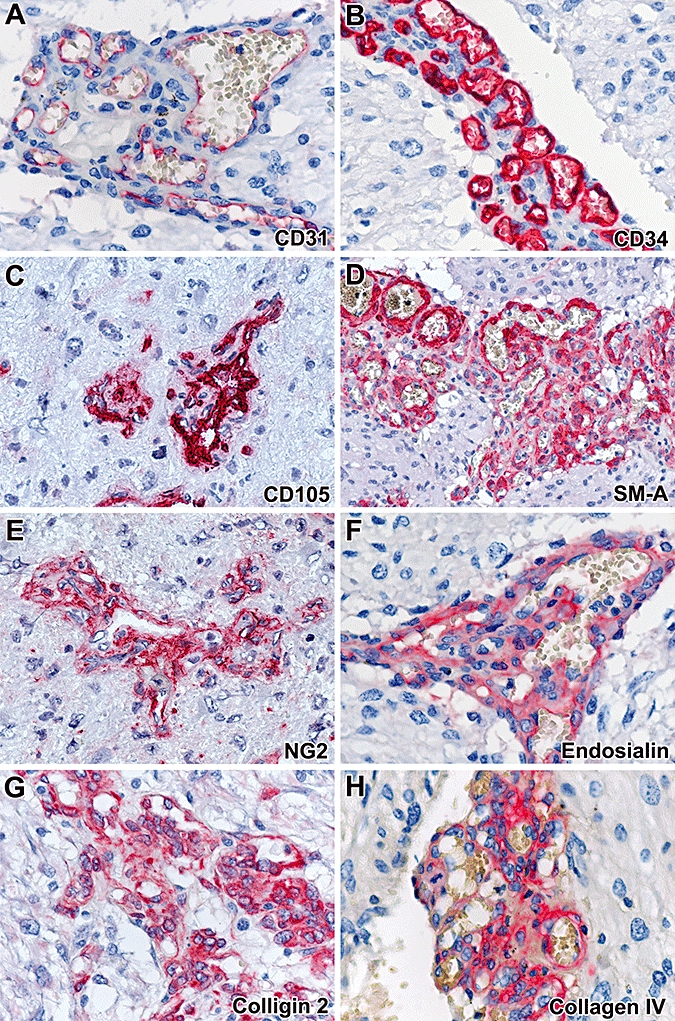
Expression of the various markers in glomeruloid blood vessels in glioma. CD31, CD34 and CD105 are expressed in the endothelial layer. SM-A, Ng2, endosialin, colligin 2 and collagen IV are expressed in all components of the vessels (panels **A–C,E–H**: ×40; **D**: ×20).

**Figure 6:**
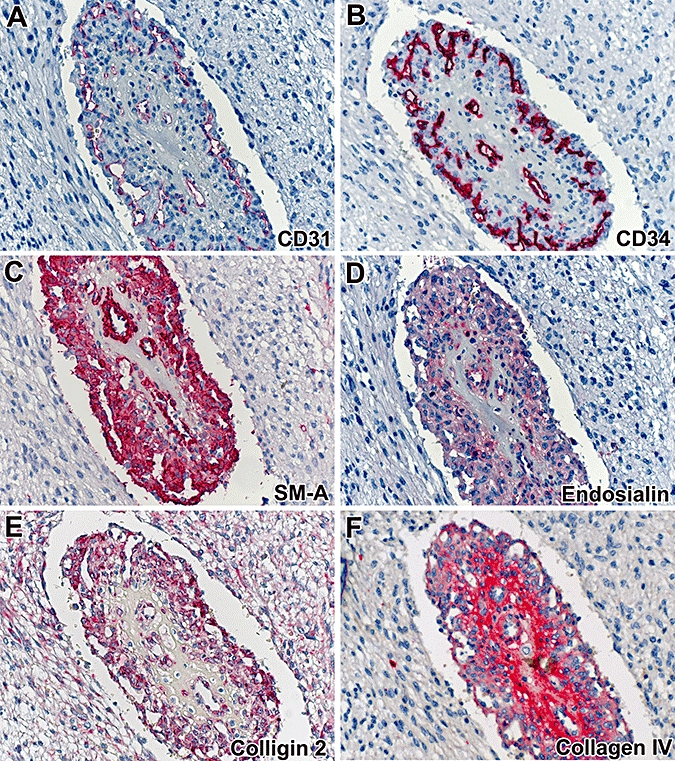
*Expression of the various markers in recanalized vessels in glioma*. CD31 and CD34 are expressed in the endothelial layer. SM-A, endosialin, colligin 2 are expressed by the surrounding cells. Collagen IV is expressed throughout the vessel wall (all panels ×20).

The vessels encountered in the glioma samples were divided in: small vessels including capillaries, which did not show morphological changes; vessels with hypertrophied walls (either with organized layering as in normal larger blood vessels or with disorganized, haphazardly arranged cellular components); vessels with glomeruloid appearance and vessels with signs of recanalization. The lumina-lining cells of capillaries in glioma were invariably positive for CD31, CD34 and CD105 (Figure 2; Table 4). In addition, there was immunopositivity for αSMA, endosialin and NG2. Expression of colligin 2 was found in all small blood vessels (Table 4). In the vessels with hypertrophied walls with organized layering, the endothelium expressed CD31 and CD34 while the intermediate and the external layers expressed αSMA and NG2 (Table 4). The inner (endothelial) and outer layer of these vessels expressed endosialin and CD105 while cells in between these two layers remained negative for these two markers. Colligin 2 was expressed in all cell layers of the hypertrophied blood vessels (Figure 3). In the hypertrophied vessels with disorganized arranged cellular components, the internal diameter of the lumen appeared irregular while the external diameter of the vessel wall varied per segment of the vessel. The endothelium stained positive for CD31, CD34 and CD105 but not for αSMA, NG2 or endosialin (Table 4). The abluminal cells of these vessels stained positive for CD105, αSMA, NG2 and endosialin. Colligin 2 was expressed in all layers of the vessel walls (Figure 4). In the glomeruloid blood vessels, the cells are haphazardly arranged around multiple small lumina. The lumina-lining cells of the glomeruloid blood vessels were positive for CD31, CD34 and CD105. The cells in the abluminal position stained positive for endosialin, NG2, and αSMA (Table 4). The expression of colligin 2 was positive in all cellular components of these blood vessels (Figure 5). The lumina-lining cells of the larger recanalized vessels were positive for CD31, CD34 and CD105 (Table 4). The other cells stained positive for αSMA, endosialin and colligin 2. Remarkably, a minority of cells in the recanalized thrombi remained negative for all markers used (Figure 6).

**Table 4:** Immunostaining of blood vessels in glioma. Abbreviation: ND = not determined.

Glioma blood vessel subtype	Layers	CD31	CD34	CD105	NG2	Endosialin	αSMA	Colligin 2
Small, normal-looking vessels	Single	+	+	+	+	+	+	+
Layered/organized hypertrophied vessels	Inner	+	+	+	−	+	−	+
	Middle	−	−	−	+	−	+	+
	Outer	−	−	+	+	+	+	+
Un-layered/ disorganized hypertrophied vessels	Inner	+	+	+	−	−	−	+
	Middle	−	−	+	+	+	+	+
	Outer	−	−	+	+	+	+	+
Glomeruloid vessels	Inner	+	+	+	+	+	+	+
	Other layers	−	−	+	+	+	+	+
Recanalized vessels	Inner	+	+	ND	ND	+/−	+/−	+/−
	Other layer	−	−	ND	ND	+/−	+/−	+/−

### Confocal laser microscopy

**Figure 7:**
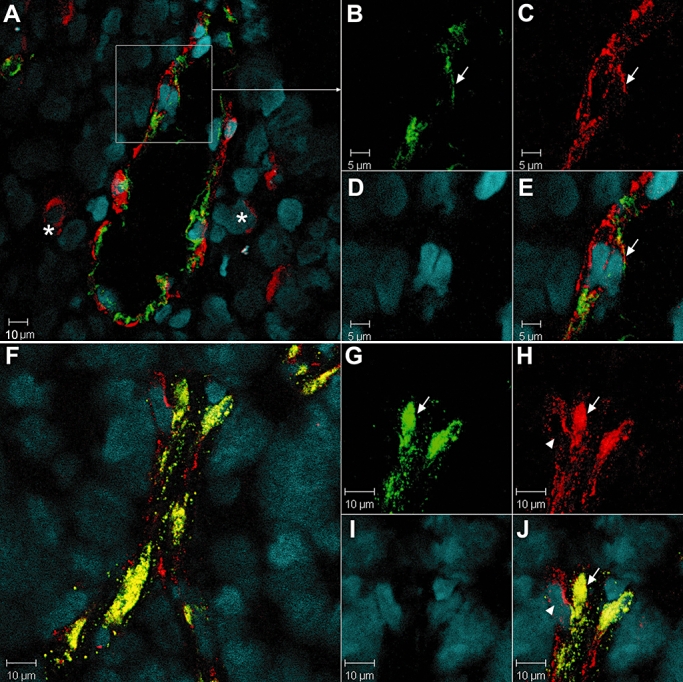
*Confocal images of glioma blood vessels visualizing the expression of colligin 2 in the endothelial cells*. **A–E.** Double immunolabeling for CD31/colligin 2 in a small blood vessel. Some CD31 expressing cells are positive for colligin 2 (arrow). Some cells around the blood vessels exclusively express colligin 2 (asterisk) [panel **B**: green = CD31; panel **C**: red = colligin 2; panel **D**: blue = 4′,6-diamidino-2-phenylindole (DAPI); panel **E**: merged picture]. **F–J.** Double immunolabeling for CD34/colligin 2 in a hypertrophied blood vessel. A complete overlap in expression of CD34 and colligin 2 in endothelial cells (arrow). Colligin 2 is also expressed by CD34-negative pericytes (panel **G**: green = CD34; panel **H**: red = colligin 2; panel **I**: blue = DAPI; panel **J**: merged image).

**Figure 8:**
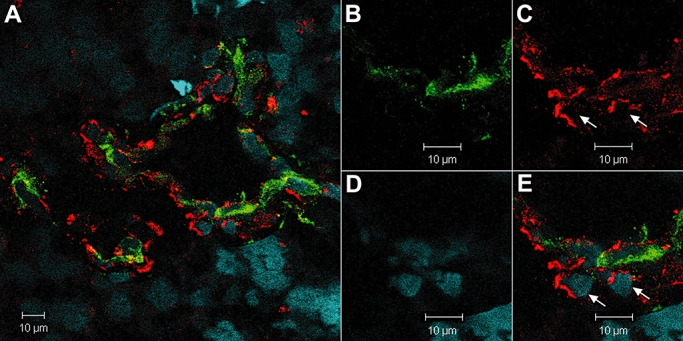
*Confocal images of glioma blood vessels for the expression of colligin 2 by activated (CD105-positive) endothelial cells*. **A–E.** Double immunolabeling for CD105/colligin 2 in hypertrophied blood vessel. CD105 and colligin 2 are expressed by endothelial cells while the cells around the endothelium express only colligin 2 (arrows) (panel **B**: green = CD105; panel **C**: red = colligin 2; panel **D**: blue = 4′,6-diamidino-2-phenylindole; panel **E**: merged picture).

The high resolution of the confocal microscope enables to detail the expression of the various markers at the level of individual cells. In all distinguished types of glioma blood vessels, the lumina-lining endothelial cells were positive for CD31 and CD34, while none of the other cellular components were positive for these markers (Figure 7). CD105, a marker for activated endothelial cells, was expressed by the endothelial cells in all different types of blood vessels in the glioma samples (Figure 8). The percentage of endothelial cells that expressed colligin 2 varied between the blood vessels in the same sample and ranged between high-, and hardly any expression. In some endothelial cells, expression levels of colligin 2 was the same as that of CD31 or CD34, while other cells showed a very low level of expression.

**Figure 9:**
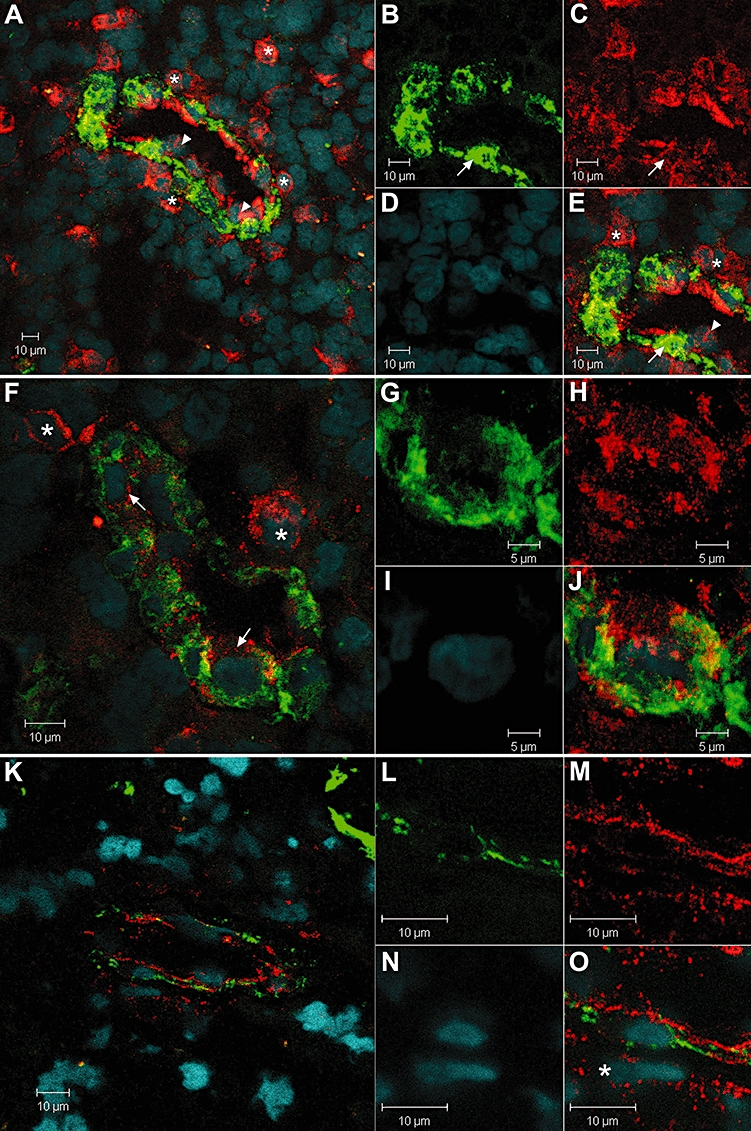
*Confocal images of glioma blood vessels for the expression of colligin 2 by the surrounding cells/pericytes*. **A–E.** Double immunolabeling for NG2/colligin 2 in hypertrophied blood vessel. Cells expressing NG2 show expression of colligin 2 (arrow). NG2 is not expressed by the endothelial cells. Some cells around the blood vessels exclusively express colligin 2 (asterix) (panel **B**: green = NG2; panel **C**: red = colligin 2; panel **D**: blue = DAPI; panel **E**: merged picture). **F–J.** Double immunolabeling for endosialin/colligin 2 in hypertrophied blood vessel. Cells expressing endosialin are positive for colligin 2 as well (arrow). Some cells around the blood vessels show exclusive expression of colligin 2 (asterix) [panel **G**: green = endosialin; panel **H**: red = colligin 2; panel **I**: blue = 4′,6-diamidino-2-phenylindole (DAPI); panel **J**: merged picture]. **K–O.** Double immunolabeling for SM-A/colligin 2 in small blood vessel. Expression of endosialin and colligin 2 in the same cells of the vessel wall. Exclusive expression of colligin 2 in cells around the blood vessels (asterisk) (panel **L**: green = endosialin; panel **M**: red = colligin 2; panel **N**: blue = DAPI; panel **O**: merged picture).

The pericytic markers NG2, endosialin and αSMA were combined with colligin 2. In all types of blood vessels, NG2 and αSMA were exclusively found in the layers around the endothelium and these cells also expressed colligin 2. Endosialin was expressed around the endothelium as well, but a low percentage of the endothelial cells of the various blood vessel subtypes also expressed this protein (Figure 9). Interestingly, scattered individual colligin 2-positive cells were present around all blood vessel subtypes. These colligin 2-expressing cells lacked expression of any of the endothelial or pericytic cell markers.

**Figure 10:**
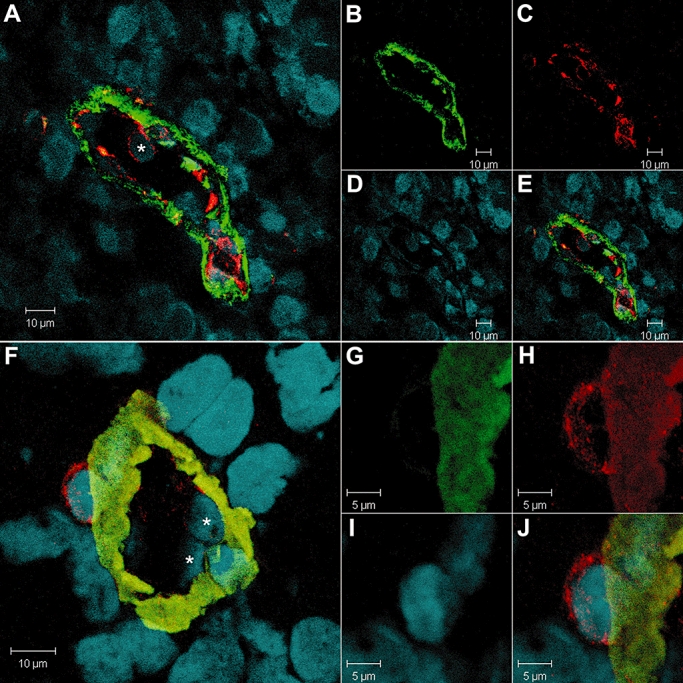
*Confocal images of glioma blood vessels for the expression of colligin 2 and collagen type I and IV*. **A–E.** Double immunolabeling for collagen type I and colligin 2 in hypertrophied blood vessel. The endothelial cells express colligin 2 only (stars), while the surrounding cells express both colligin 2 and collagen I [panel **B**: green = collagen I; panel **C**: red = colligin 2; panel **D**: blue = 4′,6-diamidino-2-phenylindole (DAPI); panel **E**: merged picture]. **F–J.** Double immunolabeling for collagen type IV and colligin 2 in a small blood vessel. Double expression of collagen IV and colligin 2 in the cells around the endothelium, while endothelial cells only express colligin 2. Exclusive expression of colligin 2 in cells around the blood vessels (stars) (panel **G**: green = collagen IV; panel **H**: red = colligin 2; panel **I**: blue = DAPI; panel **J**: merged picture).

**Figure 11:**
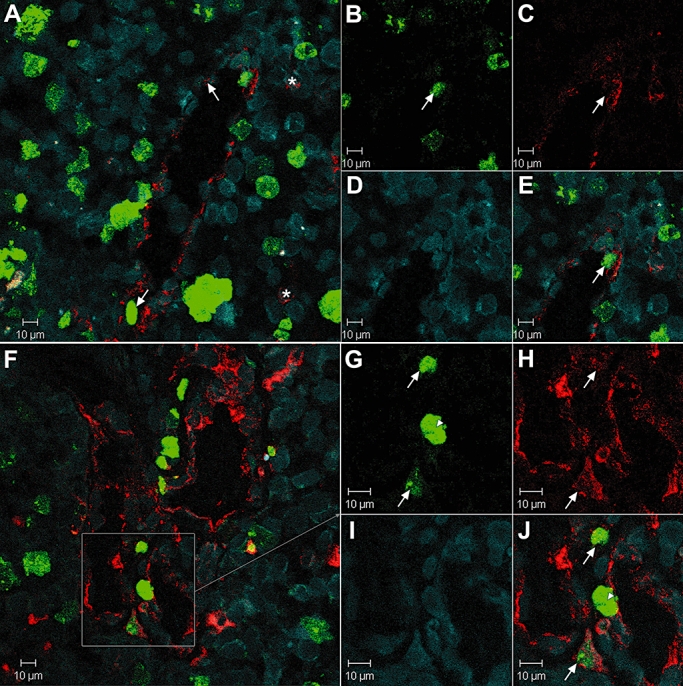
*Confocal images of glioma blood vessels for the expression of colligin 2 in Mib-1 positive cells*. **A–E.** Double immunolabeling for Mib-1/colligin 2 in a small blood vessel. A fraction of colligin 2 positive endothelial cells are Mib-1 positive (arrows) [panel **B**: green = collagen I; panel **C**: red = colligin 2; panel **D**: blue = 4′,6-diamidino-2-phenylindole (DAPI); panel **E**: merged picture]. **F–J.** Double immunolabeling for Mib-1/colligin 2 in glomeruloid vessels in glioma. Some Mib-1 positive endothelium-surrounding cells express colligin 2 (arrows) while others do not (stars) (panel **G**: green = Mib-1; panel **H**: red = colligin 2; panel **I**: blue = DAPI; panel **J**: merged picture).

**Figure 12:**
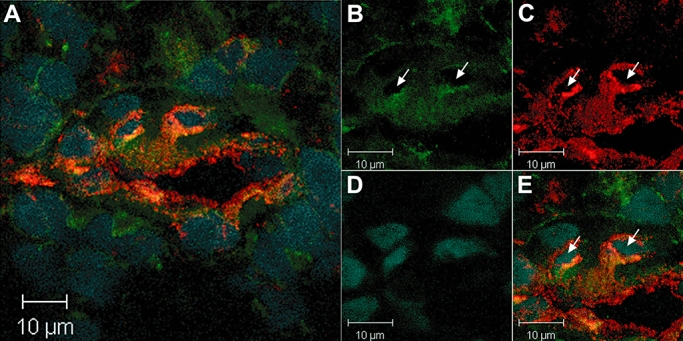
*Confocal images of glioma blood vessels for the expression of colligin 2 and glial fibrillary acidic protein (GFAP)*. **A–E.** Few GFAP positive cells in hypertrophied blood vessels express colligin 2 (arrows) (panel **B**: green = GFAP; panel **C**: red = colligin 2; panel **D**: blue = DAPI; panel **E**: merged picture).

Because colligin 2 is the chaperon for the collagen types I and IV, we included these proteins in our investigations. There appeared to be an overlap in expression of colligin 2 and collagen type I and IV. However, not all colligin 2-positive cells showed expression of collagen. For instance, some cells in the glomeruloid and small blood vessels exclusively expressed colligin 2 (Figure 10). The endothelial cells remained negative for collagens I and IV. Noticeably, the individual colligin 2-expressing cells found around the blood vessels never expressed the two collagens (Figure 10). The proliferation-related marker Mib-1 was combined with colligin 2 and co-expression was seen in some endothelial cells of small and hypertrophied vessels. In the glomeruloid vessels Mib-1 expression was seen in both colligin 2 positive and negative cells (Figure 11). The glial fibrillary acidic protein (GFAP)-positive astrocytes residing outside the blood vessels remained negative for colligin 2, but a very low percentage of these cells participating in the glioma blood vessel formation were found to co-express colligin 2 (Figure 12).

## DISCUSSION

In our previous work, we discovered that colligin 2 is expressed in glioma vasculature but not in the blood vessels of normal brain (21). The aim of the present study was to investigate which subset of vessels and which cellular components of the glioma neovasculature express colligin 2. The results show that colligin 2 was expressed at various stages of glioma blood vessels: in new vascular sprouts; in hypertrophied vessels; in glomeruloid vessels; and also in end-stage and thrombosed vessels in which recanalization is taking place. Moreover, the expression of colligin 2 was discovered in capillaries and some larger vessels that had not yet undergone any morphological change. Because colligin 2 was expressed in active angiogenesis but was not seen in blood vessels of normal brains it may be considered as an early marker for angiogenesis in glioma vasculature that remains present throughout the life cycle of the vessels.

At the level of single cells, colligin 2 is present in the lumina-lining endothelial cells showing co-expression of the endothelial markers CD31, CD34 and CD105 (Table 5). As CD105 is a marker for activated endothelial cells, colligin 2 seems to be linked with endothelial activation and active angiogenesis. Colligin 2 is also expressed by pericytic or mural cells characterized by immunopositivity for αSMA, NG2 and endosialin (Table 5). In normal human development, it has been shown that endothelial cells are driven and guided by migrating pericytes during organization of the growing vessel walls (33). The immunohistochemical profiles that are the basis for the distinction of the various cells in the glioma blood vessels like endothelial cells or pericytes are less consistent than suggested in the literature so far. The lumina-lining cells of normal brain vasculature invariably express CD31 and CD34 while the expression of the pericytic marker αSMA varies between vessels of comparable sizes (Table 3). Alpha SMA is expressed in all types and sizes of blood vessels of glioma although its expression is limited to only few blood vessels in the normal brain samples. The markers NG2, endosialin and colligin 2 were all exclusively found in glioma blood vessels but never in the blood vessels in the normal brain samples. As reported in the literature, endosialin is expressed in pericytes of glioma tissue (28). The present results confirm the specificity of endosialin for glioma vasculature and that endosialin is present in pericytes, but also show that endosialin is present in some endothelial cells (Figure 9). To a lesser extent, NG2 showed an overlap in expression with endothelial markers and appeared to be more specific for pericytic cells. The exclusive expression of colligin 2 and endosialin in morphologically normal blood vessels in glioma is illustrative of a shift in protein expression patterns prior to morphological changes. Interestingly, using confocal microscopy we found a subset of cells in brain tissue close to, but not in contact with, glioma blood vessels to express colligin 2. These cells did not express any of the endothelial/pericytic markers used in this study. It may well be that these cells are migrating vascular precursor cells on their way to merge into the vessel walls, or, alternatively, become de novo blood vessels. This is compatible with colligin 2 expression reflecting an early stage of vascular development. Further exploration as to the lineage and origin of these cells is indicated.

**Table 5:** Confocal laser microscopy of glioma blood vessels.

Endothelial cells		Pericytes	
Positive	Negative	Positive	Negative
CD31	NG2	NG2	CD31
CD34	αSMA	Endosialin	CD34
CD105		αSMA	CD105 +/−
Colligin 2		Colligin 2	
Endosialin +/−			

+/− shows varies results depending on the type of blood vessel.

Colligin 2 (also known as CBP2 or heat shock protein 47) is a collagen-binding stress protein localized in the endoplasmic reticulum (14). In the normal situation the expression of HSP47 precedes that of collagen, and in various cell types and tissues the co-expression of the proteins is present (26). The expression of HSP47 and several types of collagen is induced under pathological conditions (29). Colligin 2 is essential for the maturation of collagen by assisting in the correct folding and supporting the transportation of collagen to the basal membrane. The absence of colligin 2 causes defective maturation of the microfibrills of collagen type I and IV and impaired formation of the basal membrane (18). Colligin 2 knockout mice did not survive beyond 11.5 days post-fertilization and displayed abnormally orientated epithelial tissues and ruptured blood vessels (22). Inactivation of the expression of colligin 2 seriously affects the function of the basal membrane of blood vessels (17). Our results confirm the anticipated overlap in expression of colligin 2 with collagen types I and IV in the glioma blood vessels, corroborating the significance of colligin 2 for the formation of the basal membrane. The fact that expression of colligin 2 precedes the expression of collagen may well explain absent co-expression in some of the vascular cells examined in this study.

Resistance of brain tumors to therapy may in part be caused by the abnormal functioning of tumor vasculature caused by pathological changes of the BBB. Normally, astrocytic end-feet and basal membrane substance of the cerebral blood vessels both contribute to the BBB. The majority of blood vessels in glioma loses contact with astrocytic end-feet and loses normal anatomy and function. Disruption of the BBB was noticed in our investigations. In glioma vasculature, glial cells may participate in glioma angiogenesis (so-called “mosaic vessels”) (10), but normal formation of the end-feet for a normal BBB function is absent. In this study, by double labeling colligin2 and GFAP, we excluded the expression of colligin 2 by astrocytes in glioma tissue, but we encountered scattered astrocytes in the glioma blood vessels that expressed GFAP and colligin 2. This finding indicates that some astrocytes or astrocytic tumor cells that contribute to glioma blood vessels switch their protein expression repertoire to take part in angiogenesis. GFAP-/colligin 2-positive cells were never found in glioma tissue and they were very rarely present in the glioma blood vessels. Nevertheless, this finding is important when considering vascular cell populations for therapeutic intervention. The properties and functions of the glial cells in the mosaic vessels need further investigation.

In conclusion, we confirm that colligin 2 is expressed in glioma vasculature and we found that its expression is by the distinct cell compartments of the tumor vasculature, whether they are activated endothelial cells or cells with immunoprofiles of pericytes. In addition, scattered cells without the immunophenotype of endothelial or pericytic cells were also immunopositive for colligin 2, as were some GFAP-positive cells in some of the vessel walls. Because colligin 2 is detected in a spectrum from morphologically normal to severely disfigured glioma blood vessels, it potentially may become in use as a marker for active angiogenesis or serve as a link to targeted anti-angiogenic strategies.
